# Association Between Lower-Limb Fractures and Carpal Tunnel Syndrome: A Nationwide Population-Based Cohort Study

**DOI:** 10.3390/healthcare13222879

**Published:** 2025-11-12

**Authors:** Chun-Hui Chang, Hao-Yu Tseng, Wen-Tien Wu, Ru-Ping Lee, Jen-Hung Wang, Kuang-Ting Yeh

**Affiliations:** 1 School of Medicine, Tzu Chi University, Hualien 970374, Taiwan; 2 Department of Orthopedics, Hualien Tzu Chi Hospital, Buddhist Tzu Chi Medical Foundation, Hualien 970473, Taiwan; 3 Institute of Medical Sciences, Tzu Chi University, Hualien 970374, Taiwan; 4 Department of Medical Research, Hualien Tzu Chi Hospital, Buddhist Tzu Chi Medical Foundation, Hualien 970473, Taiwan; paulwang@tzuchi.com.tw; 5 Graduate Institute of Clinical Pharmacy, Tzu Chi University, Hualien 970374, Taiwan

**Keywords:** carpal tunnel syndrome, lower-limb fractures, assistive devices, population-based cohort study, Propensity score matching

## Abstract

**Highlights:**

**What are the main findings?**

- Lower-limb fractures increase the risk of developing carpal tunnel syndrome (CTS).
- The risk is higher in males and younger adults (20–65 years).

**What is the implication of the main finding?**

- CTS risk should be considered when prescribing assistive devices after fractures.
- Early monitoring may help prevent CTS in high-risk patients.

**Abstract:**

**Background:** Lower-limb fractures often require prolonged use of assistive devices, which may increase mechanical stress on the upper extremities. However, the association between lower-limb fractures and subsequent carpal tunnel syndrome (CTS) remains unclear. **Methods:** This nationwide population-based cohort study used Taiwan’s National Health Insurance Research Database (2011–2019) to identify 10,140 patients with lower-limb fractures and 10,140 propensity score-matched controls. Cox regression analysis estimated CTS risk after adjusting for demographics and comorbidities. **Results:** Patients with lower-limb fractures demonstrated increased CTS risk compared to controls (adjusted hazard ratio [HR] = 1.12, 95% confidence interval [CI]: 1.003–1.26; *p* = 0.044), with stronger associations in males (HR = 1.28, 95% CI: 1.05–1.55) and younger adults aged 20–65 years (HR = 1.19, 95% CI: 1.03–1.38). **Conclusions:** Lower-limb fractures are associated with modestly increased CTS risk, particularly in males and younger patients. Though biologically plausible, this observational study cannot establish causality. Heightened clinical awareness may be warranted, though prospective validation is needed.

## 1. Introduction

Lower-limb fractures are among the most common injuries encountered in daily life and clinical practice and affect individuals across all age groups and activity levels. During the recovery period, patients are typically advised to use assistive devices such as crutches, walkers, or wheelchairs to maintain mobility while minimizing weight bearing on the injured limb. Although these aids are essential for rehabilitation and provide varying degrees of stability and independence, they impose substantial biomechanical demands on the upper extremities, particularly compressive forces and repetitive loading on the wrists and hands. Studies using instrumented assistive devices have demonstrated that hand loads during cane-assisted walking can reach up to 30% of body weight [1], while grip forces on walker handles range from 5–6% of body weight during ambulation [2]. Biomechanical analysis using three-dimensional motion capture and instrumented forearm crutches has demonstrated high superior joint forces at the wrist, elbow, and shoulder during crutch-assisted gait, with loading patterns that may be asymmetrical and vary based on individual biomechanics [3]. Previous studies have demonstrated that the prolonged use of mobility aids may lead to significant biomechanical alterations in the upper limbs. For instance, the chronic use of walkers has been associated with ulnar deviation and progressive changes in wrist joint angles [4]. These findings underscore the potential adverse effects of assistive devices on upper extremity musculoskeletal structures and nerve function.

Despite these observations, there remains a paucity of research examining the prevalence and risk factors of upper-limb disorders in patients recovering from lower extremity fractures. Most existing literature has concentrated primarily on surgical management and rehabilitation protocols for lower-limb injuries, with considerably less attention devoted to secondary complications affecting the upper limbs resulting from prolonged assistive device use. This knowledge gap represents a significant clinical concern, as upper-limb dysfunction can substantially impair patients’ quality of life, independence in activities of daily living, and overall rehabilitation outcomes.

Several studies have explored upper-limb pain and overuse syndromes in long-term assistive device users, particularly among individuals with chronic conditions such as poliomyelitis [5]. One notable study demonstrated a direct association between forearm crutch use and the development of carpal tunnel syndrome (CTS) in the weight-bearing wrist [6]. Furthermore, ergonomic modifications of walker handles have been proposed as potential interventions to reduce mechanical loading on the wrist joints [7]. These findings suggest that assistive device use may contribute to upper extremity pathology through sustained biomechanical stress. However, comprehensive population-based studies investigating the relationship between lower-limb fractures and subsequent CTS remain limited.

CTS is the most prevalent peripheral entrapment neuropathy worldwide, caused by compression of the median nerve as it traverses the carpal tunnel at the wrist. Owing to the anatomical distribution of the median nerve, patients characteristically experience numbness, pain, paresthesia, or weakness affecting the palmar aspect of the thumb, index finger, middle finger, and the radial side of the ring finger. While electrodiagnostic testing with nerve conduction studies remains the gold standard for confirming CTS diagnosis, characteristic clinical symptoms and provocative signs, including Tinel’s sign and Phalen’s test, are also clinically valuable and may be sufficient for diagnosis in many cases [8,9]. Since its first clinical description in 1854 by Sir James Paget, extensive research has identified multiple risk factors for CTS development. A large-scale population-based study in Taiwan identified several significant risk factors, including female sex, previous wrist injuries, obesity, gout, and rheumatoid arthritis [10]. Additionally, systemic conditions such as Sjögren’s syndrome and end-stage renal disease have been linked to increased CTS risk [11,12]. Collectively, accumulated evidence suggests that mechanical strain, repetitive microtrauma, inflammatory conditions, and systemic metabolic diseases contribute to CTS pathogenesis. Given the substantial and sustained mechanical demands placed on the wrist during mobility aid use following lower-limb fractures, it is biologically plausible that rehabilitation-related activities and assistive device use could represent underrecognized and clinically important risk factors for CTS development.

However, no large-scale epidemiological studies have systematically examined this association. We specifically focused on CTS as the primary outcome given its status as the most common entrapment neuropathy, its well-established diagnostic criteria, and its characteristic association with repetitive wrist loading. Other upper extremity neuropathies, such as ulnar neuropathy at the elbow or radial nerve compression, were not examined in this study but may warrant investigation in future research. Therefore, we conducted this nationwide population-based cohort study to investigate whether patients with lower-limb fractures have an increased risk of developing CTS over an extended post-fracture follow-up period of up to 7 years and to identify potential demographic and clinical subgroups that may be particularly vulnerable to this complication.

## 2. Materials and Methods

### 2.1. Study Design and Data Source

This retrospective cohort study utilized data from the Taiwan National Health Insurance Research Database (NHIRD), which contains comprehensive medical records of over 99% of the Taiwanese population. The Taiwan National Health Insurance (NHI) program is a single-payer, mandatory health insurance system established in 1995, providing universal coverage for outpatient visits, inpatient care, prescription medications, and diagnostic procedures. We analyzed a representative cohort of 2,000,124 individuals to investigate the association between lower-limb fractures (LLFxs) and the subsequent development of CTS.

### 2.2. Study Population

From the representative cohort, we identified all adult patients (≥20 years) diagnosed with hip, acetabular, or peri-knee fractures between 1 January 2010 and 31 December 2018. Lower-limb fractures were identified using the ICD-9-CM codes 808 (pelvis fracture), 820 (femoral neck fracture), 821 (other femoral fractures), and 823 (tibia and fibula fractures), and the ICD-10-CM codes S32 (pelvis fracture), S72 (femur fracture), and S82 (tibia and fibula fractures). The index date was defined as the date of the first LLFx diagnosis during the study period. We excluded patients with pre-existing CTS, defined as any CTS diagnosis (ICD-9-CM code 354.0; ICD-10-CM codes G56.00, G56.01, G56.02) within one year prior to the index fracture date, to ensure only incident cases were included.

**Figure 1:**
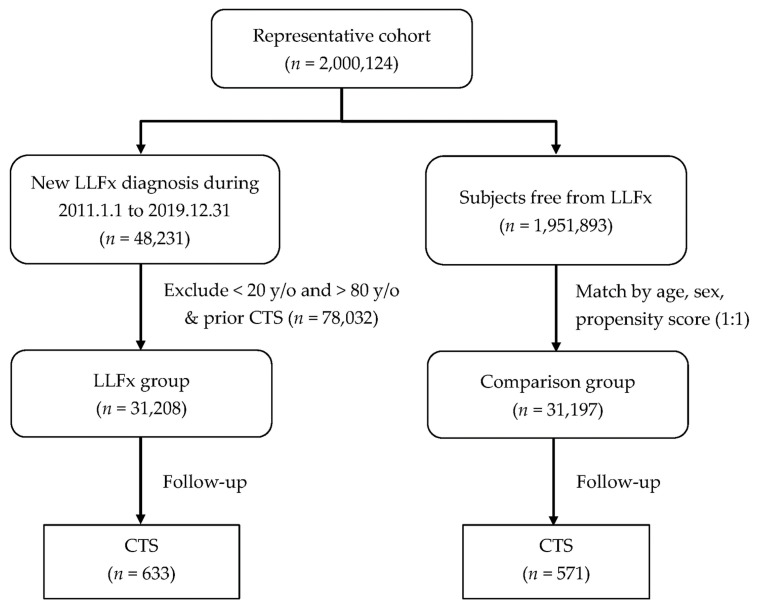
Flowchart of Study Population Selection and Matching Process. LLFx, lower-limb fracture; CTS, carpal tunnel syndrome.

Additionally, we excluded patients with any prior wrist or forearm injury to minimize confounding from pre-existing upper extremity trauma. Wrist injuries were identified using ICD-9-CM codes 813 (radius and ulna fracture), 814 (carpal bone fracture), 833 (wrist dislocation), 834 (finger dislocation), 842 (wrist sprain), and 881–887 (open wounds of hand and wrist), and ICD-10-CM codes S52–S53 (forearm fracture and dislocation), S56 (forearm muscle injury), and S60–S69 (wrist and hand injuries including fractures, dislocations, sprains, open wounds, and amputations). Patients aged < 20 or >80 years at the index date were also excluded. The upper age limit of 80 years was established to reduce confounding from multiple comorbidities, differential assistive device use, increased diagnostic uncertainty, and survival bias that are particularly prominent in very elderly populations. A flowchart of the study population selection is shown in Figure 1. After applying inclusion and exclusion criteria, the final LLFx group comprised 31,208 patients, of whom 633 developed CTS during follow-up.

### 2.3. Control Group Selection

The comparison group consisted of participants from the same cohort who did not experience LLFx during the study period (*n* = 1,951,893). Controls were matched 1:1 with the LLFx group according to age, sex, and propensity score, resulting in 31,197 matched controls. The index date for controls was assigned as the matched date corresponding to their paired LLFx patient’s fracture date. In the control group, 571 patients developed CTS during follow-up (Figure 1).

### 2.4. Propensity Score Matching

To minimize selection bias and confounding factors, we performed 1:1 propensity score matching between patients with LLFx and controls. The propensity score was calculated using logistic regression incorporating baseline demographic characteristics (age, sex, urbanization level, and geographic region) and comorbidities assessed within one year prior to the index date. While the NHIRD does not contain detailed occupational or socioeconomic data, our exclusion of all prior upper-limb injuries and inclusion of relevant comorbidities help minimize confounding from pre-existing upper extremity conditions. Comorbidities included hypertension (HTN; ICD-9-CM codes 401–405; ICD-10-CM codes I10–I13, I15), diabetes mellitus (DM; ICD-9-CM code 250; ICD-10-CM codes E10, E11, E13), hyperlipidemia (ICD-9-CM code 272; ICD-10-CM code E78), coronary artery disease (CAD; ICD-9-CM codes 410–414; ICD-10-CM codes I20–I22, I24, I25), cerebrovascular accident (CVA; ICD-9-CM codes 430–438; ICD-10-CM codes I60–I69, G45, G46), chronic liver disease (ICD-9-CM code 571; ICD-10-CM codes K70, K73, K74, K754, K760, K769, K7581, K7689), chronic renal failure (ICD-9-CM code 585; ICD-10-CM codes N184–N186, N189), depression (ICD-9-CM codes 311, 2962, 2963, 3004; ICD-10-CM codes F30, F32, F40, F41, F43, F44, F68), and rheumatoid arthritis (RA; ICD-9-CM code 714; ICD-10-CM codes M05, M06, M08, M12). Matching was performed using a caliper width of 0.2 standard deviations of the logit of the propensity score to ensure adequate balance between groups.

### 2.5. Outcome Measurement and Validation

The primary outcome was the development of incident CTS during the follow-up period. CTS was identified using ICD-9-CM code 354.0 and ICD-10-CM codes G56.00, G56.01, and G56.02. To enhance diagnostic specificity and reduce the likelihood of miscoding, we required at least two outpatient visits with CTS diagnosis codes or one inpatient diagnosis during the follow-up period. In the Taiwan NHI system, CTS diagnosis and subsequent treatment (whether conservative management or surgical intervention) require clinical documentation and physician evaluation. Reimbursement policies incentivize accurate diagnostic coding, as inappropriate coding may result in claim rejection. While individual-level clinical validation data such as nerve conduction study results are not directly accessible in the NHIRD, and specific validation studies for CTS diagnosis codes in this database are limited, validation studies of other neurological and musculoskeletal diagnoses in the NHIRD have generally demonstrated reasonable accuracy, with positive predictive values typically ranging from 70% to 90% when appropriate case definitions are applied. We acknowledge that any non-differential misclassification would likely bias our results toward the null hypothesis. The large-scale population-based design, comprehensive coverage, and our stringent case definition support reliable outcome ascertainment. The follow-up period began 30 days after the index date (to establish temporal sequence and reduce surveillance bias) and ended at the earliest of: (1) CTS diagnosis, (2) death, (3) withdrawal from the NHI program, or (4) 31 December 2019. The incidence rate was calculated per 1000 person-years.

### 2.6. Statistical Analysis

Continuous variables are presented as mean ± standard deviation, and categorical variables are presented as frequencies and percentages. After 1:1 propensity score matching, the baseline characteristics between the groups were compared using chi-square tests for categorical variables and independent *t*-tests for continuous variables. The standardized mean difference (Cohen’s d) was calculated to assess the effect size and balance between the groups after matching, with a standardized mean difference of <0.1 indicating adequate balance. Cox proportional hazards regression models were used to estimate the hazard ratios (HRs) and 95% confidence intervals (CIs) for CTS development. Univariate analysis was performed, followed by multivariate analysis adjusting for all baseline characteristics. The proportional hazards assumption was assessed using Schoenfeld residuals. In addition, competing risk analysis was conducted using the Fine–Gray Subdistribution hazard model to account for mortality during the follow-up period, treating death as a competing event. Subgroup analyses were performed using Cox regression models stratified by age group (20–65 vs. 65–80 years) and sex to explore potential effect modifications. Interaction terms were tested to assess whether the association between LLFx and CTS differed significantly across subgroups. Kaplan–Meier survival curves were constructed to visualize the cumulative incidence of CTS between the groups, and the log-rank test was used to compare survival distributions. All statistical analyses were performed using SAS software (version 9.4; SAS Institute, Cary, NC, USA) and Stata (version 16; StataCorp, College Station, TX, USA). Statistical significance was determined at a *p*-value < 0.05.

## 3. Results

### 3.1. Baseline Characteristics

After 1:1 propensity score matching, the final study cohort included 62,394 participants (31,197 in each group) (Figure 1). The baseline characteristics of the patients are summarized in Table 1. The matching process successfully balanced the baseline characteristics between the groups, with all standardized mean differences (SMD) < 0.1, indicating adequate matching quality. The mean age was 52.23 ± 17.21 years, with no significant difference between the LLFx group (52.17 ± 17.21 years) and control group (52.29 ± 17.22 years, *p* = 0.386, SMD = −0.01). The age distribution showed that 70.6% of participants were aged 20–65 years and 29.4% were aged 65–80 years, with a balanced distribution between the groups (*p* = 0.312, SMD = −0.05). Female participants comprised 53.2% of the total cohort with a similar sex distribution between the groups (*p* = 0.694, SMD = 0.00). The distribution of comorbidities was well-balanced between groups, including HTN (28.4%, *p* = 0.210), DM (17.2%, *p* = 0.603), hyperlipidemia (16.8%, *p* = 0.585), CAD (6.6%, *p* = 0.439), cerebrovascular accident (6.1%, *p* = 0.376), chronic liver disease (5.3%, *p* = 0.292), chronic renal failure (3.3%, *p* = 0.061), depression (7.4%, *p* = 0.472), and RA (2.0%, *p* = 0.794). All comorbidities showed SMD values ≤ 0.02, confirming successful matching (Table 1). The LLFx group had a significantly shorter mean follow-up time compared to controls (3.43 ± 2.20 vs. 3.53 ± 2.21 years, *p* < 0.001, SMD = −0.05). This difference likely reflects higher mortality rates in patients with lower-limb fractures. However, our use of Cox proportional hazards regression appropriately accounts for this differential follow-up duration by analyzing time-to-event data and censoring participants at their last observation date. Additionally, competing risk analysis accounting for death as a competing event yielded consistent results, confirming that differential follow-up did not substantially bias our estimates.

### 3.2. Incidence of CTS

During the follow-up period, 633 patients (2.0%) in the LLFx group and 571 patients (1.8%) in the control group developed CTS (Table 1). When comparing these proportions directly, the difference showed marginal statistical significance (*p* = 0.076, SMD = 0.01). However, this simple proportion comparison considers only the cumulative proportion of events at the end of follow-up without accounting for differences in follow-up duration or the timing of event occurrence. When expressed as incidence rates using person-years as the denominator, the LLFx group demonstrated a higher CTS incidence compared to controls (5.9 vs. 5.2 per 1000 person-years) based on 107,027 and 110,164 person-years of follow-up, respectively (Table 2). The incidence rate ratio (IRR = 1.13) closely corresponds to the hazard ratio obtained from Cox regression analysis, demonstrating consistency between time-adjusted measures. Cox proportional hazards regression, which appropriately accounts for varying follow-up durations and evaluates differences in the rate of event occurrence throughout the entire follow-up period, serves as our primary analytical approach for assessing the association between lower-limb fractures and CTS risk.

### 3.3. Risk Analysis

As presented in Table 2, univariate Cox regression analysis revealed that patients who experienced LLFx had a significantly increased risk of developing CTS compared to controls (crude HR, 1.12; 95% CI: 1.003–1.26, *p* = 0.043). After adjusting for all baseline characteristics, including age, sex, and comorbidities, in the multivariate model, the association remained statistically significant (adjusted HR, 1.12; 95% CI: 1.003–1.26, *p* = 0.044). The consistency between crude and adjusted hazard ratios reflects the successful balance of confounding variables achieved through propensity score matching.

### 3.4. Subgroup Analysis

The subgroup analysis results are presented in Table 3, revealing important variations in the association between LLFx and CTS risk across different demographic groups. Age-stratified analysis demonstrated that the association was primarily driven by younger patients, with those aged 20–65 years showing a significantly increased risk of CTS following LLFx in both crude (HR: 1.19, 95% CI: 1.04–1.35, *p* = 0.011) and adjusted models (HR: 1.19, 95% CI: 1.04–1.35, *p* = 0.012). In contrast, no significant association was observed in patients aged 65–80 years in either crude (HR: 0.95, 95% CI: 0.76–1.20, *p* = 0.686) or adjusted models (HR: 0.95, 95% CI: 0.76–1.20, *p* = 0.680). Sex-stratified analysis revealed differential effects between male and female patients. Male patients demonstrated a significantly elevated risk of CTS after LLFx in both crude (HR: 1.30, 95% CI: 1.06–1.60, *p* = 0.011) and adjusted models (HR: 1.31, 95% CI: 1.07–1.61, *p* = 0.009). However, female patients showed no significant association in either crude (HR: 1.05, 95% CI: 0.92–1.20, *p* = 0.489) or adjusted models (HR: 1.05, 95% CI: 0.92–1.20, *p* = 0.488). Interaction tests showed borderline significance for both age (*p* = 0.110) and sex (*p* = 0.083), suggesting potential effect modification by these demographic factors.

### 3.5. Survival Analysis

**Figure 2:**
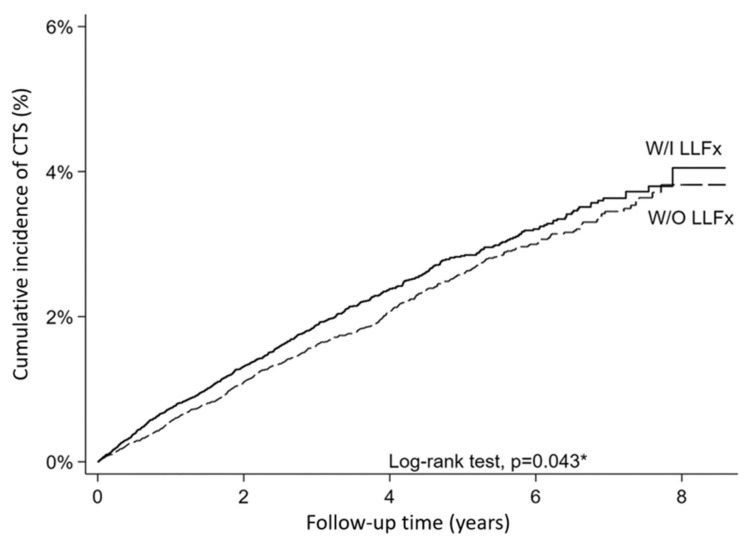
Cumulative incidence of carpal tunnel syndrome in patients with and without lower-limb fractures. LLFx, lower-limb fracture; CTS, carpal tunnel syndrome. * *p*-value < 0.05 was considered statistically significant after test.

The Kaplan–Meier survival curves in Figure 2 demonstrate the cumulative incidence of CTS in the LLFx and control groups following 1:1 propensity score matching (*n* = 62,394). The curves showed consistent separation throughout the follow-up period, with the LLFx group exhibiting a higher cumulative CTS incidence than the control group. Early divergence occurs and persists over time, indicating a sustained increase in the risk of CTS in patients with lower-limb fractures. The log-rank test confirmed statistically significant differences in the survival distributions between the two groups (*p* = 0.041), with a steeper trajectory in the LLFx group, supporting the Cox regression findings of an elevated hazard ratio.

## 4. Discussion

In this nationwide population-based cohort study, we demonstrated that patients with lower-limb fractures had a modest but statistically significant increase in the risk of developing CTS compared with propensity-score-matched controls. The adjusted hazard ratio of 1.12 (95% CI: 1.003–1.26, *p* = 0.044) indicates a statistically significant but modest increase in CTS risk, suggesting that fracture-related rehabilitation and the use of assistive devices may represent potential contributors to upper-limb morbidity that warrant further investigation.

While the overall adjusted hazard ratio of 1.12 represents a modest relative risk increase, this finding carries substantial clinical significance. Given the high incidence of lower-limb fractures (approximately 300–400 per 100,000 person-years), even a 12% relative risk increase translates into thousands of potentially preventable CTS cases annually. More importantly, our subgroup analyses revealed substantially stronger associations in high-risk populations: males (HR = 1.28, 95% CI: 1.05–1.55) and younger adults aged 20–65 years (HR = 1.19, 95% CI: 1.03–1.38)—the economically active population for whom CTS has particularly significant occupational implications. Unlike many established CTS risk factors, the proposed mechanism involving excessive upper extremity loading represents a potentially modifiable risk factor amenable to ergonomic interventions and prophylactic strategies. Numerous established occupational health guidelines for preventing work-related musculoskeletal disorders are based on similar relative risks in the 1.1–1.3 range. As the first large-scale population-based study examining this relationship, our findings provide the epidemiological foundation for developing targeted prevention strategies and designing future intervention trials.

Previous studies have identified obesity, occupational factors, and comorbidities such as HTN, DM, hypothyroidism, uremia requiring dialysis, and RA as risk factors for CTS [10,13,14]. Therefore, in our 1:1 propensity score-matched cohort study, we included these comorbidities as matching variables to isolate the independent association between lower-limb fractures with subsequent assistive device use and CTS risk. Baseline demographic characteristics, including female sex and age, are well-established risk factors for CTS. Alterations in hormone levels are considered one of the key pathophysiological mechanisms that cause CTS in females [15,16]. CTS may result from elevated intracarpal pressure due to either a smaller carpal canal or an increased volume of the structures within it [17]. In Taiwan, females and middle-aged individuals (50–59 years) have been reported to have a higher incidence of CTS [10]. Considering both statistical power and clinical relevance, there were too few cases of some comorbidities (e.g., RA [2.0%] and chronic kidney disease [3.3%]) to allow for a meaningful stratified analysis. In contrast, age and sex are immutable demographic characteristics with direct clinical significance and may modify the effect of assistive device use on CTS risk. Therefore, we conducted exploratory subgroup analyses focusing on these two variables. However, these subgroup findings should be interpreted with caution, as the interaction tests did not reach statistical significance (age: *p* = 0.110; sex: *p* = 0.083), and the analyses were not pre-specified as primary hypotheses. These results are hypothesis-generating and require validation in future studies specifically designed to examine effect modification.

In the subgroup analysis, the association was more pronounced in younger patients (20–65 years old) and in males, whereas no significant association was observed in older patients (65–80 years old) or females. Although the interaction tests did not reach statistical significance (*p* = 0.110 for age and *p* = 0.083 for sex), these borderline *p*-values suggest potential effect modifications worthy of further investigation. These findings may be hypothetically attributed to the possibility that individuals in the younger age group, particularly men, constitute the main productive workforce and may have higher levels of physical activity; thus, they may have greater dependence on mobility aids after fractures to maintain their daily activities and occupational demands. However, as activity level and occupational exposure were not directly measured in our database, this interpretation remains speculative and requires validation in future studies with prospective assessment of these variables. In addition, male patients may experience higher wrist stress potentially related to greater body weight and differences in the manner in which crutches or walkers are used, though sex-specific biomechanical data and device usage patterns were not directly measured in our study. These findings are consistent with prior clinical observations and biomechanical evidence, suggesting that the use of lower-limb assistive devices increases the risk of developing CTS. Previous studies have shown that the long-term use of mobility aids, such as walkers and crutches, alters wrist biomechanics, including increased ulnar deviation and changes in joint angles, thereby elevating the mechanical stress on the carpal tunnel [5,6,7]. Previous research has demonstrated that postsurgical patients using a walker experience increased stress in their upper extremity joints [18]. This finding may help explain the mechanism linking lower-limb fractures to the subsequent incidence of CTS. Recent biomechanical investigations quantified these stresses [19,20]. For example, instrumented crutch and walker systems have revealed that hand and wrist joint loads increase substantially during partial weight-bearing gait. Although these loads are generally below the acute physiological thresholds (not the same as standard guidelines) reported in the literature, repetitive high loading may still lead to long-term nerve compression or joint problems. Notably, these findings were primarily derived from healthy participants (mean age approximately 36–38 years, with a male-to-female ratio of 1:1), which only partially overlaps with the characteristics of our study population [19,20]. Similarly, reviews on human–mobility aid interaction emphasize that repetitive loading, ulnar deviation, and altered wrist kinematics are consistent contributors to CTS pathogenesis among assistive device users [21,22]. Based on a recent meta-analysis, upper-limb complications in patients with lower-limb fractures should be considered as more important and warrant greater clinical attention [23]. Previous studies have demonstrated that different types of crutches can cause varying degrees of stress in the upper extremities [19]. Our study provides supporting epidemiological evidence confirming an association between lower-limb fractures and subsequent CTS development. Therefore, orthopedic surgeons and rehabilitation specialists should counsel patients at a high risk of upper-limb complications to select ergonomically optimized assistive devices that minimize wrist loading. Regular clinical surveillance of CTS symptoms should be implemented to enable early diagnosis and timely intervention to preserve hand function and quality of life in high-risk patients.

It is noteworthy that while the cumulative incidence difference between groups reached only marginal statistical significance when compared as simple proportions (*p* = 0.076), time-to-event analysis using the Cox proportional hazards model revealed a statistically significant association (adjusted HR = 1.12, 95% CI: 1.003–1.26, *p* = 0.044). This discrepancy reflects fundamental differences in analytical approaches: simple proportion comparisons assess only the cumulative burden of events at study end without considering when events occurred, whereas Cox regression evaluates the rate of event occurrence throughout the entire follow-up period and incorporates individual variations in follow-up duration. Given the differential follow-up time between groups (3.43 vs. 3.53 years, *p* < 0.001), the timing of CTS events contributed meaningfully to the observed association. The consistency between our incidence rate ratio (5.9 vs. 5.2 per 1000 person-years, IRR = 1.13) and the hazard ratio (HR = 1.12) further supports the validity of our time-to-event analysis. This methodological consideration underscores the importance of employing appropriate statistical methods that account for temporal dynamics in longitudinal cohort studies.

### 4.1. Clinical Recommendations

Based on our findings, several clinical recommendations can be made to mitigate the risk of CTS in patients recovering from lower-limb fractures. Orthopedic surgeons and rehabilitation specialists may consider implementing systematic risk stratification at the time of fracture diagnosis, with particular attention to potentially high-risk subgroups, specifically males aged 20–65 years with anticipated prolonged assistive device use. However, it should be noted that these subgroup findings are based on exploratory analyses with borderline interaction *p*-values (age: *p* = 0.110; sex: *p* = 0.083), and therefore, should be considered provisional recommendations that require validation in future prospective studies. A baseline upper extremity assessment may be beneficial before initiating mobility aid use, particularly in patients with pre-existing risk factors for CTS. When prescribing assistive devices, clinicians should consider prioritizing ergonomically optimized options that theoretically minimize wrist loading, such as platform crutches or walkers with adjustable handles that maintain a neutral wrist position [19]. This recommendation is based on biomechanical principles and ergonomic theory rather than direct clinical trial evidence demonstrating reduced CTS incidence. While biomechanical studies have shown that different assistive device designs produce varying degrees of upper extremity stress [19], prospective clinical trials evaluating whether ergonomic modifications effectively prevent CTS development are still needed. Appropriate fitting, height adjustment, and comprehensive patient education regarding correct usage techniques may theoretically help reduce abnormal wrist posture and cumulative mechanical stress, though the effectiveness of these interventions in preventing CTS has not been formally evaluated in clinical trials.

For potentially high-risk patients, regular clinical surveillance for early CTS symptoms may be considered as part of routine follow-up protocols, though the cost-effectiveness and clinical benefit of systematic screening have not been established. Clinicians may consider inquiring about nocturnal paresthesia, numbness in the median nerve distribution, and hand weakness during each visit. Simple provocative tests, such as Tinel’s sign and Phalen’s test, may be considered for patients reporting symptoms, though the sensitivity and specificity of these tests for early CTS detection in this specific population have not been established. When symptoms develop, timely interventions, including activity modification, nighttime wrist splinting in a neutral position, and referral for electrodiagnostic studies, may be considered based on standard CTS management guidelines. A multidisciplinary approach involving orthopedic surgeons, physiatrists, and therapists may be beneficial for optimal management and prevention of upper-limb complications, though the specific effectiveness of such approaches in this population requires further investigation.

### 4.2. Strengths and Limitations

Studies on poliomyelitis survivors and chronic crutch users have similarly reported an increased incidence of CTS [5,6]. However, most previous research has been limited to specific populations or small sample sizes [24]. The major strengths of our study include its large sample size, nationwide population-based coverage, long-term follow-up period, and 1:1 propensity score matching design, which effectively minimized confounding by demographic and comorbidity factors. Using a comprehensive national database enabled us to capture a representative cohort with sufficient statistical power to detect moderate associations.

Nonetheless, several limitations should be acknowledged. First, and most critically, this study lacks detailed data on assistive device exposure. The NHIRD does not contain information on whether patients used assistive devices, device type (crutches, walkers, canes), duration or intensity of use, or temporal relationship between device use and CTS onset. Consequently, the presumed mechanism linking lower-limb fractures to CTS through increased upper extremity loading remains inferential and hypothesis-generating rather than empirically demonstrated. While biologically plausible and supported by biomechanical studies [16,17], our observational design cannot definitively establish this causal pathway. Without objective measurements, we cannot establish dose–response relationships or determine optimal CTS surveillance timing. Alternative mechanisms (systemic inflammation, immobilization effects, shared risk factors) cannot be excluded. Stronger associations in males and younger patients may reflect unmeasured differences in activity levels or device usage patterns.

Second, the NHIRD lacks information on fracture severity, rehabilitation protocols, and occupational/lifestyle factors. Importantly, many unmeasured variables—particularly assistive device characteristics and fracture severity—likely function as mediators rather than confounders in the causal pathway. Our observed HR (1.12) represents the total effect encompassing pathways mediated through assistive device use, suggesting intervention targets (device selection, usage training, wrist protection) could reduce CTS risk. However, formal mediation analysis was not possible with available data. Our analysis treated lower-limb fractures as a composite exposure to maximize statistical power, acknowledging this does not capture heterogeneity across fracture sites differing in severity and device usage requirements. However, all lower-limb fractures share the common feature of requiring upper-extremity-dependent mobility assistance. Stratifying by fracture site would substantially reduce power given the modest effect size (HR = 1.12) and low CTS incidence. Several methodological features mitigate unmeasured confounding concerns. Propensity score matching balanced socioeconomic proxies (income, urbanization) strongly correlating with occupational exposures. Our time-to-event analysis inherently captures cumulative exposure effects, with consistency between IRR (1.13) and HR (1.12) supporting temporal relationship robustness. The one-year lag period excluded pre-existing subclinical CTS and established appropriate temporal sequencing. Biological plausibility is supported by biomechanical evidence demonstrating assistive device use increases carpal tunnel pressure and alters wrist kinematics consistent with CTS pathogenesis [16,17].

Third, CTS diagnoses were identified using administrative ICD codes without electrodiagnostic confirmation, and specific validation studies for CTS diagnosis codes in the NHIRD are limited. To minimize misclassification bias, we employed a stringent case definition requiring at least two outpatient visits or one inpatient diagnosis with CTS codes. Additionally, the Taiwan NHI reimbursement system requires clinical documentation for CTS diagnosis and treatment, incentivizing accurate coding. Validation studies of other diagnoses in the NHIRD have demonstrated reasonable accuracy (positive predictive values 70–90%). Any non-differential misclassification would likely bias results toward the null, suggesting our findings may underestimate the true association. However, an important concern is differential detection bias: patients with lower-limb fractures likely have more frequent healthcare encounters during their recovery period, which may increase opportunities for CTS diagnosis compared to matched controls, even if the true incidence of CTS is similar. This surveillance bias could partially account for the observed association. While we implemented a 30-day lag period to exclude immediate post-fracture diagnoses and conducted competing risk analyses to account for differential follow-up patterns, these measures may not completely eliminate detection bias. Future studies with standardized, protocol-driven screening for CTS in both exposed and unexposed groups would help clarify whether the observed association reflects true increased incidence or differential detection.

Fourth, the NHIRD does not systematically capture detailed occupational categories or individual-level socioeconomic indicators. To address this limitation, we excluded all patients with prior wrist or forearm injuries, incorporated urbanization level and geographic region as proxy measures, and included multiple comorbidities in our propensity score matching that may serve as indirect indicators of lifestyle factors. However, residual confounding from unmeasured occupational and socioeconomic factors remains possible.

Fifth, our upper age limit of 80 years, established to reduce confounding from multiple comorbidities, differential assistive device use, and diagnostic uncertainty, limits generalizability to very elderly populations.

Sixth, while our subgroup analyses revealed differential associations by age and sex, these findings are exploratory in nature. The interaction tests did not reach conventional statistical significance (age: *p* = 0.110; sex: *p* = 0.083), and these analyses were not pre-specified in our original study protocol. Therefore, these subgroup findings should be considered hypothesis-generating and require confirmation in future studies with adequate statistical power to detect effect modification.

Finally, although the association was statistically significant and consistent across sensitivity analyses, the effect size was modest (adjusted HR = 1.12, 95% CI: 1.01–1.25). This may reflect heterogeneity in assistive device usage patterns, unmeasured confounding, or the multifactorial nature of CTS etiology. However, given the high prevalence of lower-limb fractures in aging populations, even modest relative increases in CTS risk may translate to substantial absolute numbers of affected individuals, warranting clinical attention and further investigation.

### 4.3. Future Directions

Future research should address these limitations through several complementary approaches. Prospective cohort studies with detailed documentation of assistive device types, duration of use, and weight-bearing patterns are needed to establish dose–response relationships between mobility aid usage and CTS development. Randomized controlled trials comparing different assistive devices with ergonomic modifications could provide evidence-based guidance for device selection in clinical practice. Additionally, investigating the effectiveness of preventive interventions, such as wrist splinting protocols, nerve gliding exercises, and structured patient education programs, in reducing the incidence of CTS among patients with lower-limb fractures would have significant clinical implications. Biomechanical studies using motion analysis and pressure sensors can further elucidate the mechanisms linking specific assistive device use patterns to carpal tunnel pathologies. The objective sensor-based measurements are also recommended for further investigation. For example, a twin-axis wrist goniometer could measure the wrist deviation while using a walking assistant [25]. Future studies could compare the finding on the objective measurements with the clinical finding of new-onset upper-limb impairment to find the direct correlation between using different walking aids and the new-onset upper-limb disorders. Finally, cost-effectiveness analyses evaluating screening and prevention strategies for high-risk subgroups could inform resource allocation and clinical guideline development.

## 5. Conclusions

Our study identified an association between lower-limb fractures and a modest but statistically significant increased risk of CTS, with the association being more pronounced in younger patients (aged 20–65 years) and males. While the proposed mechanism involving increased upper extremity loading during assistive device use is biologically plausible, our observational design cannot establish causality, and alternative or contributing mechanisms cannot be excluded. These findings suggest that patients recovering from lower-limb fractures, particularly younger individuals and men, may warrant heightened clinical awareness for CTS symptoms. Future prospective studies with objective measurements of assistive device exposure and wrist biomechanics are essential to validate the proposed mechanism and establish causal relationships. If confirmed, these findings could inform the development of targeted prevention strategies and guide evidence-based assistive device selection to minimize upper extremity complications during lower-limb fracture recovery.

## Figures and Tables

**Table 1:** Demographics.

Variables	1:1 Propensity Score Matching (*n* = 62,394)				
	Control	LLFx	Total	*p*-Value	SMD
N	31,197	31,197	62,394		
Age	52.29 ± 17.22	52.17 ± 17.21	52.23 ± 17.21	0.386	−0.01
Age group	-	-	-	0.312	−0.05
20–65 y/o	21,966 (70.4%)	22,082 (70.8%)	44,048 (70.6%)		
65–80 y/o	9231 (29.6%)	9115 (29.2%)	18,346 (29.4%)		
Sex	-	-	-	0.694	0.00
Male	14,577 (46.7%)	14,627 (46.9%)	29,204 (46.8%)		
Female	16,620 (53.3%)	16,570 (53.1%)	33,190 (53.2%)		
Hypertension (%)	8916 (28.6%)	8774 (28.1%)	17,690 (28.4%)	0.210	−0.01
Diabetes (%)	5390 (17.3%)	5340 (17.1%)	10,730 (17.2%)	0.603	0.00
Hyperlipidemia (%)	5273 (16.9%)	5221 (16.7%)	10,494 (16.8%)	0.585	0.00
Coronary artery disease (%)	2039 (6.5%)	2088 (6.7%)	4127 (6.6%)	0.439	0.01
Cerebrovascular accident (%)	1937 (6.2%)	1883 (6.0%)	3820 (6.1%)	0.376	−0.01
Chronic liver disease (%)	1623 (5.2%)	1683 (5.4%)	3306 (5.3%)	0.292	0.01
Chronic renal failure (%)	974 (3.1%)	1058 (3.4%)	2032 (3.3%)	0.061	0.02
Depression (%)	2333 (7.5%)	2285 (7.3%)	4618 (7.4%)	0.472	−0.01
Rheumatoid arthritis (%)	602 (1.9%)	612 (2.0%)	1214 (2.0%)	0.794	0.00
CTS (%)	571 (1.8%)	633 (2.0%)	1204 (1.9%)	0.076	0.01
Follow-up time (yr.)	3.53 ± 2.21	3.43 ± 2.20	3.48 ± 2.20	<0.001 *	−0.05

Data are presented as n or mean ± standard deviation. * *p*-value < 0.05 was considered statistically significant after test. Abbreviations: LLFx, lower-limb fracture; SMD, standardized mean differences; CTS, carpal tunnel syndrome.

**Table 2:** Risk of CTS between patients with and without LLFx (*n* = 62,394).

Variables	1:1 Propensity Score Matching (*n* = 62,394)	
	LLFx	
	No	Yes
Patient numbers	31,197	31,197
CTS cases	571	633
Person-years	110,164	107,027
Incidence rate ^a^	5.2	5.9
Univariate model		
Crude HR (95% CI)	1.12 (1.003–1.26)	1
*p*-value	0.043 *	
Multivariate model ^b^		
Adjusted HR (95% CI)	1.12 (1.003–1.26)	1
*p*-value	0.044 *	

^a^ Per 1000 person-years. ^b^ Multivariate Cox proportional hazard regression model with adjustment for all baseline characteristics shown in Table 1. Abbreviations: HR, hazard ratio; CI, confidence interval; CTS, carpal tunnel syndrome. * *p*-value < 0.05 was considered statistically significant after test

**Table 3:** Subgroup analysis of risk of CTS between patients with and without LLFx (*n* = 62,394).

Variables	1:1 Propensity Score Matching (*n* = 62,394)			
	Crude HR ^a^ (95% CI)	*p*-Value	Adjusted HR ^a^ (95% CI)	*p*-Value
Main model				
No	1.00		1.00	
Yes	1.12 (1.003–1.26)	0.043 *	1.12 (1.003–1.26)	0.044 *
Age				
20–65 y/o				
No	1.00		1.00	
Yes	1.19 (1.04–1.35)	0.011 *	1.19 (1.04–1.35)	0.012 *
65–80 y/o				
No	1.00		1.00	
Yes	0.95 (0.76–1.20)	0.686	0.95 (0.76–1.20)	0.680
Sex				
Male				
No	1.00		1.00	
Yes	1.30 (1.06–1.60)	0.011 *	1.31 (1.07–1.61)	0.009 *
Female				
No	1.00		1.00	
Yes	1.05 (0.92–1.20)	0.489	1.05 (0.92–1.20)	0.488
*p* for interaction				
Age	0.110			
Sex	0.083			

^a^ Cox’s proportional hazards model. Abbreviations: HR, hazard ratio; CI, confidence interval; CTS, carpal tunnel syndrome. * *p*-value < 0.05 was considered statistically significant after test

## Data Availability

The datasets generated and analyzed during the current study are not publicly available due to the restrictions of the Taiwan National Health Insurance Research Database (NHIRD) regulations and patient privacy protection requirements. Access to NHIRD data requires approval from the Taiwan Ministry of Health and Welfare and adherence to strict data security protocols. Researchers interested in accessing similar datasets may apply through the Health and Welfare Data Science Center, Ministry of Health and Welfare, Taiwan (https://dep.mohw.gov.tw/DOS/cp-5119-59201-113.html) (accessed on 25 September 2025). The statistical code used for data analysis is available from the corresponding author upon reasonable request and with appropriate data use agreements in place.

## Abbreviations

The following abbreviations are used in this manuscript:

CAD	Coronary artery disease
CI	Confidence intervals
CTS	Carpal tunnel syndrome
DM	Diabetes mellitus
HR	Hazard ratios
HTN	Hypertension
ICD	International Classification of Diseases
LLFx	Lower-limb fractures
NHIRD	National Health Insurance Research Database
RA	Rheumatoid arthritis
SMD	Standardized mean differences
CVA	Cerebrovascular accident
